# Strategy of Developing Oral Vaccine Candidates Against Co-infection of Porcine Diarrhea Viruses Based on a *Lactobacillus* Delivery System

**DOI:** 10.3389/fmicb.2022.872550

**Published:** 2022-04-04

**Authors:** Tiantian Guo, Chong Gao, Jianhui Hao, Xiao Lu, Kun Xie, Xiaona Wang, Jiaxuan Li, Han Zhou, Wen Cui, Zhifu Shan, Yanping Jiang, Xinyuan Qiao, Lijie Tang, Li Wang, Yijing Li

**Affiliations:** College of Veterinary Medicine, Northeast Agricultural University, Harbin, China

**Keywords:** porcine viral diarrhea, mucosal immunity, *Lactobacillus casei*, oral vaccine, multi-vaccine

## Abstract

The number of co-infections with multiple porcine diarrhea viruses has increased in recent years. Inducing mucosal immunity through oral immunization is an effective approach for controlling these pathogens. To generate a multi-pathogen vaccine against viral co-infection, we employed the *Lactobacillus* vector platform, which was previously used to generate potent candidate vaccines against various diseases. Two strategies were used to test the protective efficiency of recombinant *Lactobacillus* against multiple diarrhea viruses. First, we used a mixture of recombinant *Lactobacillus* separately expressing antigens of transmissible gastroenteritis virus (TGEV), porcine epidemic diarrhea virus (PEDV), and porcine rotavirus (PoRV). Next, we used a recombinant *Lactobacillus* expressing an antigen fusion protein of the above viruses. Twenty-four newborn piglets were divided into three groups and orally immunized with a mixture of recombinant *Lactobacillus*, recombinant *Lactobacillus* expressing the antigen fusion protein, or sterile phosphate-buffered saline daily for seven consecutive days after birth. After immunization, the piglets were randomly selected from each group for oral administration of PEDV, and these piglets were then cohabited with piglets without PEDV infection for 7 days. The protective effect against PEDV was evaluated based on clinical symptoms, viral shedding, and intestinal pathological damage. Piglets immunized with recombinant *Lactobacillus* showed specific mucosal and humoral immune responses to the three viruses and were protected against severe diarrhea and intestinal pathology. Our results highlight the potential of an oral multi-pathogen vaccine based on *Lactobacillus* to prevent transmission and limit the severity of viral co-infection.

## Introduction

Viral diarrhea, caused by enterovirus infections acquired *via* mucosal surfaces, is a problem in pig farms worldwide. Viral diarrhea causes high morbidity and mortality of neonatal piglets, resulting in large economic impacts (Sun et al., 2016). The main pathogens causing porcine viral diarrhea are porcine epidemic diarrhea virus (PEDV), porcine transmissible gastroenteritis virus (TGEV), and porcine rotavirus (PoRV), and, in recent years, the newly discovered porcine deltacoronavirus, porcine cristavirus, and swine acute diarrhea syndrome coronavirus (Song et al., 2015; Zhang et al., 2019; Wen et al., 2021). Co-infection with multiple viruses has been reported in clinical samples of porcine diarrhea in recent years (Liu et al., 2013; Wang et al., 2014; Chang et al., 2016). Therefore, the development of multi-vaccines is urgently required. However, few multi-vaccines are currently available on the market, and most are inactivated or attenuated vaccines administered by injection.

The fecal-oral route is thought to be the main mode of transmission of porcine diarrheal viruses, with the intestinal villus as the primary site of infection (Kim et al., 2011; Thomas et al., 2015; Jiang et al., 2016), where an effective mucosal immune response is the first line of defense for neutralizing pathogens (Mestecky, 1987; Mcghee et al., 1992; Nirmal et al., 2014). Secretory IgA (sIgA) plays a key role against pathogen invasion at mucosal sites by causing the agglutination and neutralization of bacteria, viruses, and toxins, as well as by stimulating T and B cells, enhancing mucosal immunity (Austin et al., 2003). Most sIgA is released into the gastrointestinal fluid, saliva, tears, urine, and other secretions and is then secreted to the mucosal surface together with secretions to exert immunity-related effects (Renegar et al., 2004). Mucosal vaccination not only induces a strong sIgA response to defend against viral infection at the mucosal surface but also produces systemic serum IgG to neutralize newly generated viruses (Thomas et al., 2015). A previous study on the effects of different administration routes (oral, intranasal, and intramuscular) on systemic and mucosal immune responses induced by PEDV infection suggested that oral inoculation generates more comprehensive immune responses compared to those by the other routes (Yza et al., 2019). This may be because mucosal vaccination can be achieved *via* oral vaccination with antigens that can stimulate immune routes similar to those of viral infection (Staats et al., 1994). In addition, mucosal vaccination is needle-free, comparatively convenient, has a lower risk of causing hypersensitivity reactions, and is cost-effective (Mantis et al., 2011). Therefore, oral mucosal vaccination is a promising method for preventing porcine viral diarrhea.

Nevertheless, the bottleneck in the development of oral vaccines is that denaturation of antigens in the stomach and intestinal tract prevents them from stimulating the intestinal mucosa (Renukuntla et al., 2013). To overcome this difficulty, bacterial delivery systems that can survive in adverse environments have been used to deliver antigens to the intestinal tract (Bhuyan et al., 2018). Safety is an important factor in qualified delivery systems, and food-grade lactic acid bacteria (LAB) are an excellent platform for fulfilling this requirement (Craig et al., 2019). Most LAB are “generally recognized as safe” according to the United States Food and Drug Administration and fulfill the criteria of the Qualified Presumption of Safety notion developed by the European Food Safety Authority (Daniel et al., 2011). In addition, LAB perform numerous beneficial functions, such as improving nutrient absorption, adhering to deleterious substances, increasing the immune response, and inhibiting viral replication (Martín et al., 2010). A previous study showed that LAB can survive in the presence of bile and low pH (Pfeiler et al., 2007; Wall et al., 2007) and thus can adapt to the conditions encountered during transit through the gastrointestinal tract. Therefore, many researchers have transformed LAB into delivery vectors for live vaccines to transport heterogeneous antigens (Rajoka et al., 2017; Chiabai et al., 2019; Liu et al., 2019; Natalia et al., 2019). In most studies, oral administration of the LAB vaccine elicited both antigen-specific systemic and sIgA immune responses that could eradicate the pathogen in post-immunization pathogen challenge models (Daniel et al., 2011). We previously demonstrated that recombinant *Lactobacillus* expressing PEDV or TGEV antigens induced specific mucosal and humoral immune responses in piglets (Liu et al., 2011; Hou et al., 2018). To prevent co-infection with porcine diarrhea virus, we developed a multi-vaccine based on *Lactobacillus* in this study.

The purpose of this study was to investigate the immunogenicity of *Lactobacillus* expressing multiple antigens simultaneously and to determine whether oral *Lactobacillus* multi-vaccines can prevent viral infection. Three types of porcine diarrhea viruses (TGEV, PEDV, and PoRV) were selected as research models. Two strategies (mixed group and fused group) were adopted to explore the feasibility and effectiveness of the *Lactobacillus* multi-vaccine. We observed humoral responses in the mucosa and systemically against all three viruses in newborn piglets. The piglets were protected against viral challenge and cohabitation infection following oral immunization with recombinant *Lactobacillus casei*. Notably, the effect of the mixed group was slightly better than that of the fused group. Our results suggest that using recombinant *Lactobacillus* is a promising vaccine strategy against coinfection with multiple viruses.

## Materials and Methods

### Strain, Plasmid, and Virus

*Lactobacillus casei* ATCC 393 was kindly provided by the Netherlands NIZO Institute and grown anaerobically in de Man, Rogosa, and Sharpe (MRS) broth at 37°C without shaking. The *Lactobacillus* constitutive expression plasmid pPG-T7g10-PPT (Song et al., 2014; encoded resistance to chloramphenicol and contained an HCE promoter, PgsA anchor, T7g10 enhancer, and rrnBT1T2 terminator), *Escherichia coli* pMD19-T-6Ds/TG1, pMD19-T-COE/TG1, and pMD19-T-VP4/TG1 were constructed and preserved in our laboratory; the PEDV GT/2017 strain, belonging to the G2b subtype, was isolated from clinical samples and identified in 2017 in our laboratory; the TGEV TH-98 and PoRV JL94 strains were isolated in our laboratory.

### Animals

Twenty-four antibody-seronegative, healthy newborn piglets were purchased from the Acheng Experimental Practice Base of Northeast Agricultural University (Harbin, China). The piglets were breastfed until day 7 after birth and then fed with animal milk powder every 6 h. Animal studies were performed according to the regulations of the Animal Experiment Ethics Committee of the Northeast Agricultural University, China (review number: NEAUEC20210337).

### Plasmids and Recombinant Strain Construction

**Figure 1:**
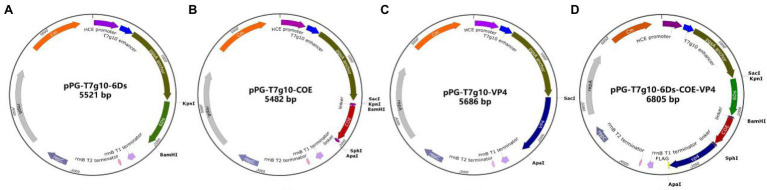
Schematic diagram showing the construction of recombinant plasmids. The fragments 6Ds, COE, VP4, and 6Ds-COE-VP4 were inserted into the vector pPG-T7g10-PPT digested by *Sac*I and *Apa*I to generate the recombinant plasmids pPG-T7g10-6Ds **(A)**, pPG-T7g10-COE **(B)**, pPG-T7g10-VP4 **(C)**, and pPG-T7g10-6Ds-COE-VP4 **(D)**.

A schematic diagram of the recombinant plasmid construction is shown in Figure 1. The details of primers used in this paper are shown in Table 1. Briefly, primers F1 and R1 were used to amplify the 6Ds gene using the plasmid pMD19-T-6Ds as a template. Primers F2 and R2 were used to amplify the VP4 gene using pMD19-T-VP4 as a template. Using plasmid pMD19-T-COE as a template, the primers F3/R3 and F4/R4 were used to amplify the COE gene, linker sequence, and *Sac*I, *Kpn*I, *BamH*I, *Sph*I, and *Apa*I sites. After digesting the vector at restriction enzyme sites *Sac*I and *Apa*I, the genes 6Ds, VP4, and COE were inserted into the expression plasmid pPG-T7g10-PPT, giving rise to recombinant plasmids pPG-T7g10-6Ds, pPG-T7g10-VP4, and pPG-T7g10-COE, respectively. The 6Ds fragment was inserted into the *Kpn*I*/BamH*I sites of 19 T-simple-COE to generate the plasmid pMD19-T-6Ds-COE. The VP4 fragment was inserted into the *Sph*I/*Apa*I sites of the recombinant plasmid pMD19-T-6Ds-COE to generate the plasmid pMD19-T-6Ds-COE-VP4. The 6Ds-COE-VP4 fragment was inserted into the *Sac*I and *Apa*I sites of the expression plasmid pPG-T7g10-PPT to generate the plasmid pPG-T7g10-6Ds-COE-VP4. All four recombinant expression plasmids were verified using polymerase chain reaction (PCR) and sequencing. Finally, the recombinant plasmids were transformed into *L. casei* 393 *via* electroporation to generate the recombinant strains pPG-T7g10-6Ds/LC393, pPG-T7g10-COE/LC393, pPG-T7g10-VP4/LC393, and pPG-T7g10-6Ds-COE-VP4/LC393.

**Table 1:** Details of primers used in this study.

Gene	ID	Primer sequences
6Ds	F1	5′-CGAGCTCATGGGTACCAGATCTTGTTAT-3′
	R1	5′-GGGCCCCTACTCGAGTCTAGAGGATCC-3′
VP4	F2	5′-CATGCATGCGCTTCGCTCATTTATAGAC-3′
	R2	5′-GGGCCCCTAAGCTCTTGTGTGCACTATCTC-3′
COE	F3	5′-**GAGGCTGCCGCAAAGGAAGCCGCCGCAAAA**GATTATAAGGATGACGATGACAAGA-3′
	R3	5′-**CTTGGCTGCAGCCTCTTTGGCTGCGGCTTC**AACGTCCGTGACACCTTCAAGTGGT-3′
	F4	5′-CGAGCTCATGGGTACCCATAAACATAAATCTAGAGAGGCTGCCGCAAAGGAAG-3′
	R4	5′-GGGCCCTTTCTACATCATGCATGC **CTTGGCTGCAGCCTCTTTGGCT**-3′
ORF3	ORF3-F	5′-TCCTAGACTTCAACCTTACG-3′
	ORF3-R	5′-GGTGACAAGTGAAGCACAGA-3′

*Restriction enzyme recognition sites used for cloning are underlined. Linkers are shown in bold*.

### Identification of Antigen Expression in Recombinant *Lactobacillus* Using Western Blot

To analyze the expression of the proteins of interest in recombinant strains pPG-T7g10-6Ds/LC393, pPG-T7g10-COE/LC393, pPG-T7g10-VP4/LC393, and pPG-T7g10-6Ds-COE-VP4/LC393, the identified strains were cultured in MRS medium containing chloramphenicol (10 mg/ml) at 37°C for 16 h and then harvested by centrifugation (Heraeus Pico17 centrifuge, Thermo Fisher Scientific, Waltham, MA, United States) at 10000 × *g* for 2 min. After cell lysis with lysozyme (10 mg/ml) and washing with sterile deionized water, the bacterial precipitate was resuspended in phosphate-buffered saline (PBS) and mixed with 5× sodium dodecyl sulfate buffer to obtain the protein samples. These samples were subjected to western blotting using mouse anti-6Ds serum, rabbit anti-COE serum and rabbit anti-VP4 serum (1:200 dilution) prepared in our laboratory. Anti-FLAG tag mouse monoclonal antibody (1:1000 dilution) was used as the primary antibody, and horseradish peroxidase (HRP)-conjugated goat anti-mouse IgG and HRP-conjugated goat anti-rabbit IgG (1:5000 dilution) were used as secondary antibodies. Finally, the target protein was detected and visualized using western enhanced chemiluminescence substrate and visualized.

### Identification of Antigen Expression in Recombinant *Lactobacillus* Using Immunofluorescence

To further evaluate the expression of the target protein, recombinant strains were cultured in MRS medium at 37°C for 16 h, and 500 μl of the culture was collected and centrifuged at 3500 × *g* for 5 min. The bacterial precipitate was washed three times with PBS, and the bacterial sediment was resuspended in mouse anti-6Ds serum, rabbit anti-COE serum, rabbit anti-VP4 serum, and anti-FLAG tag mouse monoclonal antibody (1:100 dilution) at 37°C for 1 h. Subsequently, the cells were incubated with fluorescein isothiocyanate-conjugated goat anti-mouse IgG and fluorescein isothiocyanate-conjugated goat anti-rabbit IgG (1:200 dilution) at 37°C for 45 min in the dark. The cells were visualized using fluorescence microscopy.

### Animal Grouping and Immunizing Procedure

Recombinant *Lactobacillus* strains were cultured in MRS medium for 16 h without agitation, washed with sterile PBS, and resuspended in PBS to a final concentration of 10^10^ colony-forming units (CFU)/ml. Twenty-four newborn piglets were divided into three groups. Six piglets were orally immunized with a mixture of 2 × 10^10^ CFU/ml pPG-T7g10-6Ds/LC393, pPG-T7g10-COE/LC393, and pPG-T7g10-VP4/LC393 (mixed group); six piglets were orally immunized with 2 × 10^10^ CFU/ml pPG-T7g10-6Ds-COE-VP4/LC393 (fused group), and the remaining 12 piglets were orally administered 2 ml of sterile PBS (PBS group). The immunization program was conducted daily for seven consecutive days after birth.

### Anti-TGEV/PEDV/PoRV Specific sIgA Levels of Immunized Piglets

After immunization, anal and nasal swabs of piglets were collected daily and soaked in 1 ml of cold sterile PBS at 4°C overnight. After centrifugation at 10000 × *g* for 10 min at 4°C, the supernatant was stored at −40°C. Specific sIgA levels in the samples were detected using enzyme-linked immunosorbent assay (ELISA) (Zhao et al., 2017). Briefly, polystyrene microtiter plates were coated with purified TGEV/PEDV/PoRV for 12 h at 4°C, washed three times with PBS containing 0.05% Tween-20 (PBST), blocked at 37°C for 2 h with 5% skim milk in PBST, washed with PBST, and incubated for 2 h at 37°C. After washing, commercial HRP-conjugated goat anti-porcine sIgA diluted to 1:5000 was added to the plate, incubated at 37°C for 1 h, and washed with PBST, followed by color development using 3,3′,5,5′ tetramethylbenzidine chromogenic solution at 37°C for 10 min. The reaction was stopped by adding 2 N H_2_SO_4_, and the optical density at 450 nm was determined using an ELISA reader (SpectraMax® ABS, Molecular Devices, Sunnyvale, CA, United States). Each sample was tested in triplicate.

### Anti-TGEV/PEDV/PoRV Serum-Specific IgG Levels of Immunized Piglets

Blood flow from the anterior vena cava was collected from piglets in each group on days between 0 and 7 post-immunization. Serum was prepared, and serum-specific IgG responses were measured using ELISA. The method was the same as that for sIgA, but the secondary antibody was HRP-conjugated goat anti-porcine IgG (1:5000).

### Grouping of Piglets

**Figure 2:**
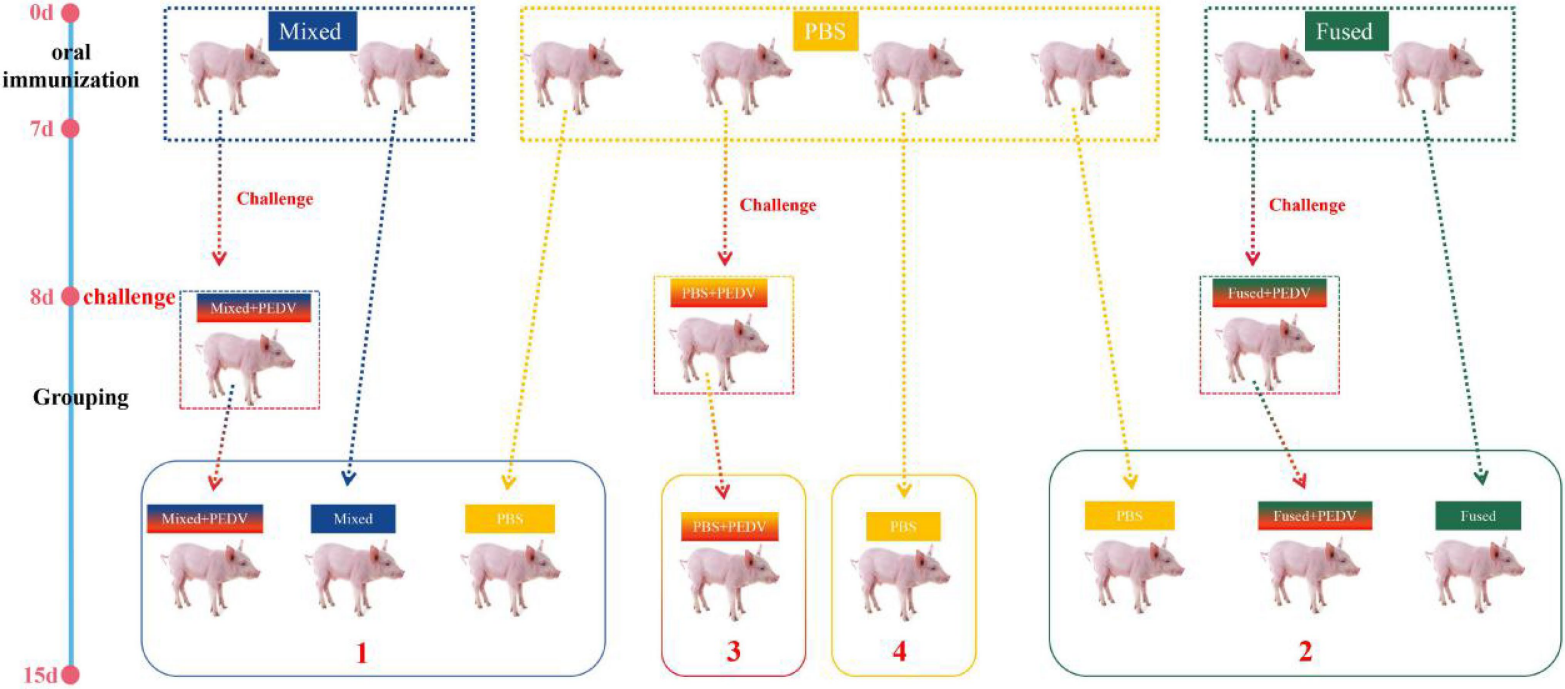
Schematic diagram of the immunization, challenge, and cohabitation of piglets. Twenty-four newborn piglets were divided into three groups for immunization. The immunization program was conducted daily for 7 consecutive days after birth. 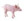 indicates three piglets. The blue dotted box indicates six piglets orally immunized with a mixture of 2 × 10^10^ CFU/ml pPG-T7g10-6Ds/LC393, pPG-T7g10-COE/LC393, and pPG-T7g10-VP4/LC393 (mixed group); the yellow dotted box indicates 12 piglets orally administered with PBS (PBS-group); and the green dotted box indicates 6 piglets orally immunized with 2 × 10^10^ CFU/ml pPG-T7g10-6Ds-COE-VP4/LC393 (fused-group). 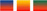 indicates three piglets randomly selected from each group after immunization for PEDV challenge on day 8. The piglets were regrouped into four different isolators (numbers 1–4) for cohabiting until they were euthanized on day 15.

After 7 days of continuous immunization, piglets were randomly selected from the mixed, fused, and PBS groups (three piglets per group) for oral administration of 4 ml PEDV (10^7^ of the median tissue culture infectious dose), and the piglets were grouped as shown in Figure 2.

### Clinical Symptoms and Weight Changes

The piglets were weighed prior to challenge and at 7 days post-challenge to calculate body weight gain, and the clinical symptoms of piglets were recorded daily and scored according to the guidelines in Table 2.

**Table 2:** Scoring system for evaluating piglets for signs of infection.

	Criteria for evaluation	Score
Diarrhea degree	Normal	0
	Soft stool	1
	Soft stool mixed with watery dung	3
	Watery diarrhea	4
Mental state	Normal	0
	Mild lethargy (slow to move, head down)	1
	Increased lethargy (lie down, occasionally stand)	3
	Severe lethargy (recumbent, moribund)	4

### Virus Shedding Through Feces of Piglets

Anal swabs were collected from the piglets daily after the challenge, and virus in the anal swabs was detected using reverse transcription PCR (RT-PCR). Briefly, one anal swab from each pig was suspended in 1 ml PBS at 4°C overnight, and the supernatant was harvested by centrifugation at 5000 × *g* for 3 min. RNA was extracted and then reverse transcribed into cDNA, and PCR was performed using primers ORF3-F and ORF3-R.

### Macroscopic Examination and Histopathology Assessment

All experimental piglets were euthanized on day 8 after challenge and dissected to observe lesions in the intestinal tract. In addition, jejunum samples were embedded in paraffin wax, and pathological sections were prepared and stained with hematoxylin and eosin.

### Statistical Analysis

The data were analyzed using GraphPad Prism 6 software (GraphPad, Inc., La Jolla, CA, United States), and all data are expressed as the mean ± standard deviation. Data were analyzed using two-way analysis of variance, with at least three independent experiments. The results were considered as significant at *p* < 0.05 and highly significant at *p* < 0.01.

## Results

### Construction of Recombinant Expression Vectors of *Lactobacillus*

To improve the expression of antigens and ensure their localization on the surface of LAB, the previously constructed expression vector pPG-T7g10-PPT was used. This vector contains the enhancer T7g10 fragment, HCE constitutive promoter, and PgsA anchor sequence (Liu et al., 2011). The protective antigen (6Ds, COE, and VP4) genes of TGEV, PEDV, and PoRV were inserted into pPG-T7g10-PPT separately or combined into the same vector (Figure 1). Four types of recombinant lactobacilli expression vectors were identified using PCR and sequencing. The sequencing results showed that the above vectors were successfully constructed.

### Expression of Viral Antigens in Recombinant *Lactobacillus*

**Figure 3:**
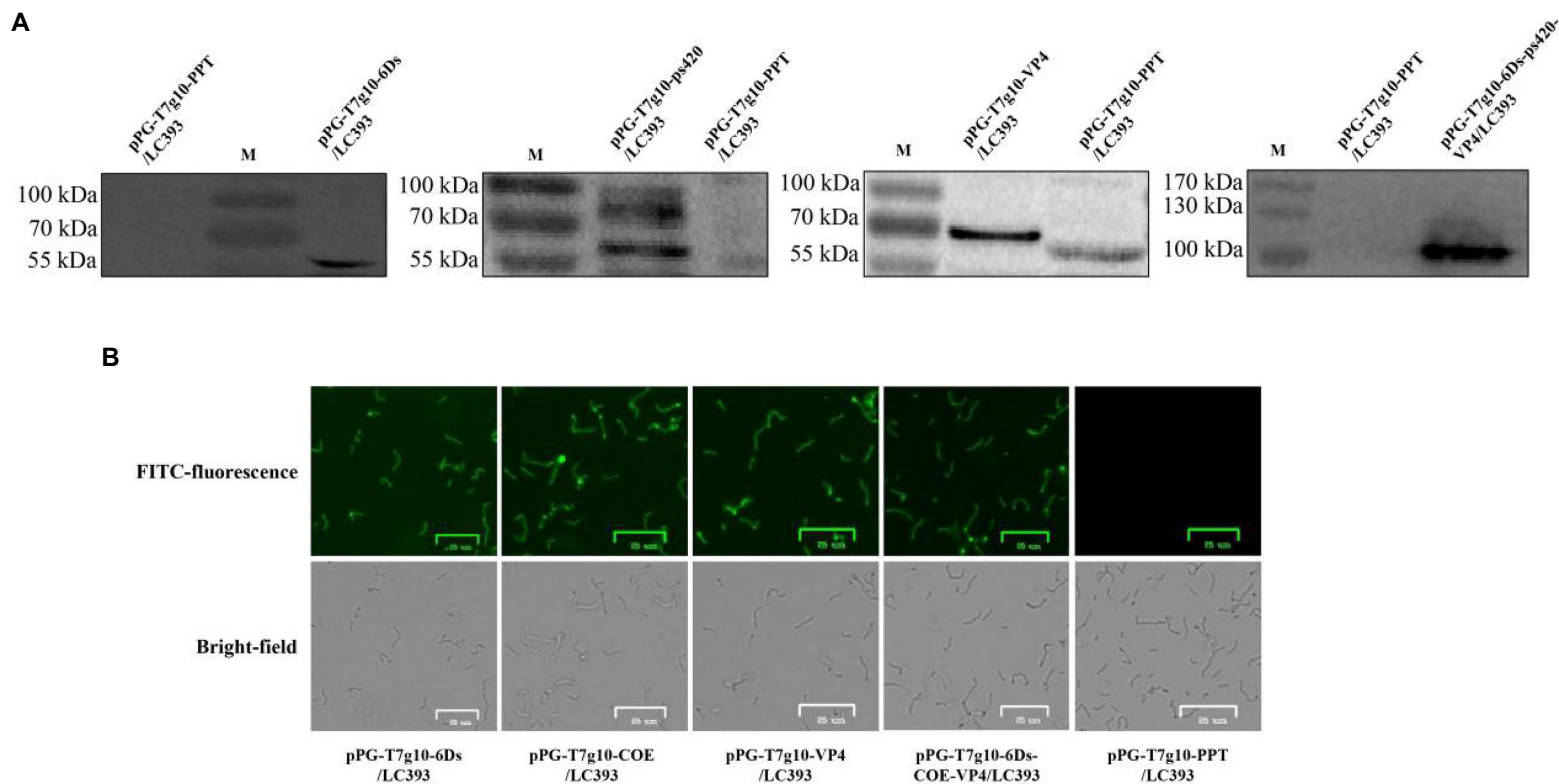
Identification of proteins of interest expressed in recombinant *Lactobacillus*. **(A)** Identification of the proteins of interest using western blotting. The relevant immunoreactive bands were detected in the cell pellets of strain pPG-T7g10-6Ds/LC393, pPG-T7g10-COE/LC393, pPG-T7g10-VP4/LC393, and pPG-T7g10-6Ds-COE-VP4/LC393 but not in those of strain pPG-T7g10-PPT/LC393. **(B)** Identification of proteins of interest using immunofluorescence assay. Green fluorescence was observed in strain pPG-T7g10-6Ds/LC393, pPG-T7g10-COE/LC393, pPG-T7g10-VP4/LC393, and pPG-T7g10-6Ds-COE-VP4/LC393 but not in strain pPG-T7g10-PPT/LC393.

Recombinant *Lactobacillus* was obtained by electroporation of the recombinant plasmid into *Lactobacillus* cells. Cell lysates of the recombinant strains were analyzed using western blotting after 16 h of culture. Correctly sized proteins were detected in all inserts of pPG-T7g10-6Ds/LC393, pPG-T7g10-COE/LC393, pPG-T7g10-VP4/LC393, and pPG-T7g10-6Ds-COE-VP4/LC393. The negative control, pPG-T7g10-PPT/LC393, did not display a corresponding immunoreactive band (Figure 3A). Immunofluorescence assays were performed to analyze the expression of the proteins of interest. As shown in Figure 3B, green fluorescence was observed for recombinant *Lactobacillus* but not for pPG-T7g10-PPT/LC393. These results indicate that the proteins of interest were successfully expressed in recombinant *Lactobacillus*.

### Determination of Anti-TGEV/PEDV/PoRV-Specific sIgA Level of Immunized Piglets

**Figure 4:**
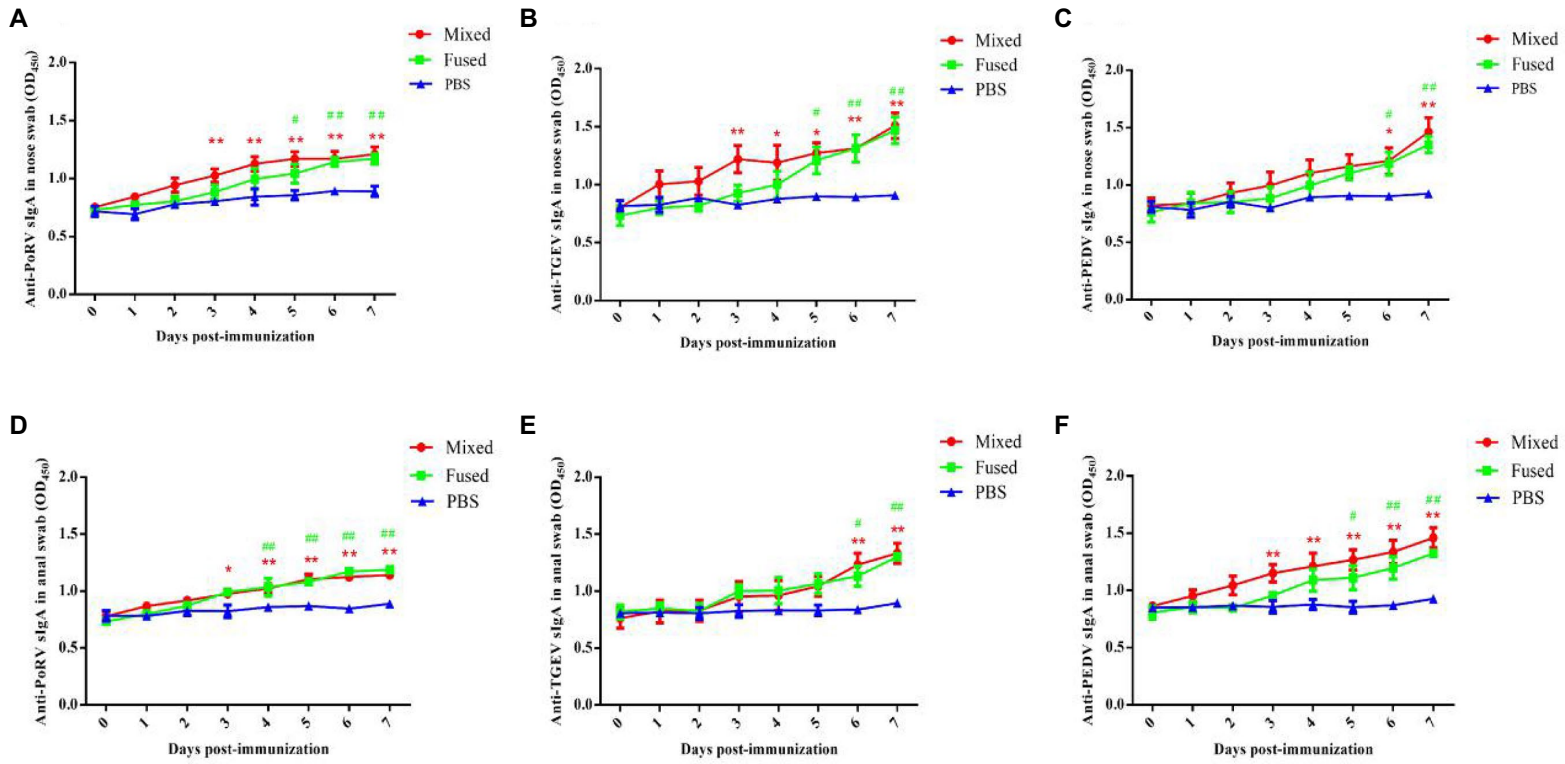
Levels of anti-TGEV/PEDV/PoRV specific sIgA in immunized piglets. The levels of anti-TGEV/PEDV/PoRV-specific sIgA was detected using ELISA. **(A)** Anti-PoRV specific sIgA in nose swab. **(B)** Anti-TGEV specific sIgA in nose swab. **(C)** Anti-PEDV specific sIgA in nose swab. **(D)** Anti-PoRV specific sIgA in anal swab. **(E)** Anti-TGEV specific sIgA in anal swab. **(F)** Anti-PEDV specific sIgA in anal swab. “*” Represents the comparison between mixed-group and PBS group, *p < 0.05, **p < 0.01. “#” represents the comparison between fused-group and PBS group, *^#^p* < 0.05, *^##^p* < 0.01.

Anti-TGEV/PEDV/PoRV-specific sIgA was assessed to evaluate the ability of recombinant *Lactobacillus* strains to induce mucosal immune responses in piglets. Anal and nasal swabs were collected from the piglets at 0–7 days post-immunization, and specific sIgA levels in the samples were detected using ELISA. As shown in Figure 4, anti-TGEV/PEDV/PoRV-specific sIgA in the nasal swabs and anal swabs post-immunization was detected in both the mixed group and fused group and gradually increased over time. No significant differences were observed in the PBS group before and after immunization. Anti-TGEV/PEDV/PoRV-specific sIgA levels in the nasal swabs and anal swabs from piglets in the mixed and fused groups were significantly higher than those in the PBS group from days 3 to 7 post-immunization (*p* < 0.05). The levels of sIgA in the mixed and fused groups did not exhibit significant differences; however, sIgA was produced earlier in the mixed group than in the fused group.

### Determination of Anti-TGEV/PEDV/PoRV-Specific IgG Level in Serum of Immunized Piglets

**Figure 5:**
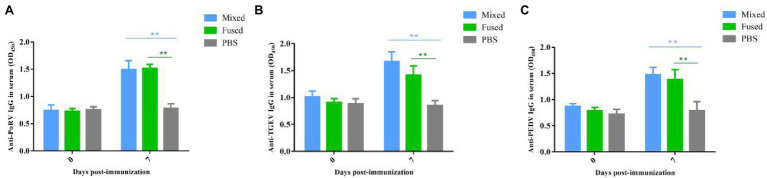
Levels anti-TGEV/PEDV/PoRV specific IgG in the serum of immunized piglets. Serum collected from piglets on days 0 and 7 post-immunization were prepared, and the serum specific IgG levels were measured using ELISA. **(A)** Anti-PoRV specific IgG in serum. **(B)** Anti-TGEV specific IgG in serum. **(C)** Anti-PEDV specific IgG in serum. **p < 0.01.

Anti-TGEV/PEDV/PoRV-specific IgG was assessed to evaluate the ability of recombinant *Lactobacillus* to induce humoral immune responses in piglets. Twenty-four newborn piglets were divided into three groups for immunization (mixed, fused, and PBS groups). Serum collected from piglets between days 0 and 7 post-immunization was prepared, and serum-specific IgG responses were measured using ELISA. As shown in Figure 5, anti-TGEV/PEDV/PoRV-specific IgG was detected at day 7 post-immunization in both the mixed and fused groups. No significant differences were observed in the PBS group before and after immunization. The levels of serum anti-TGEV/PEDV/PoRV-specific IgG in the mixed and fused groups were significantly higher than those in the PBS group (*p* < 0.01). However, there was no significant difference between the mixed and fused groups.

### Clinical Symptoms and Weight Changes of Piglets After Challenge

**Figure 6:**
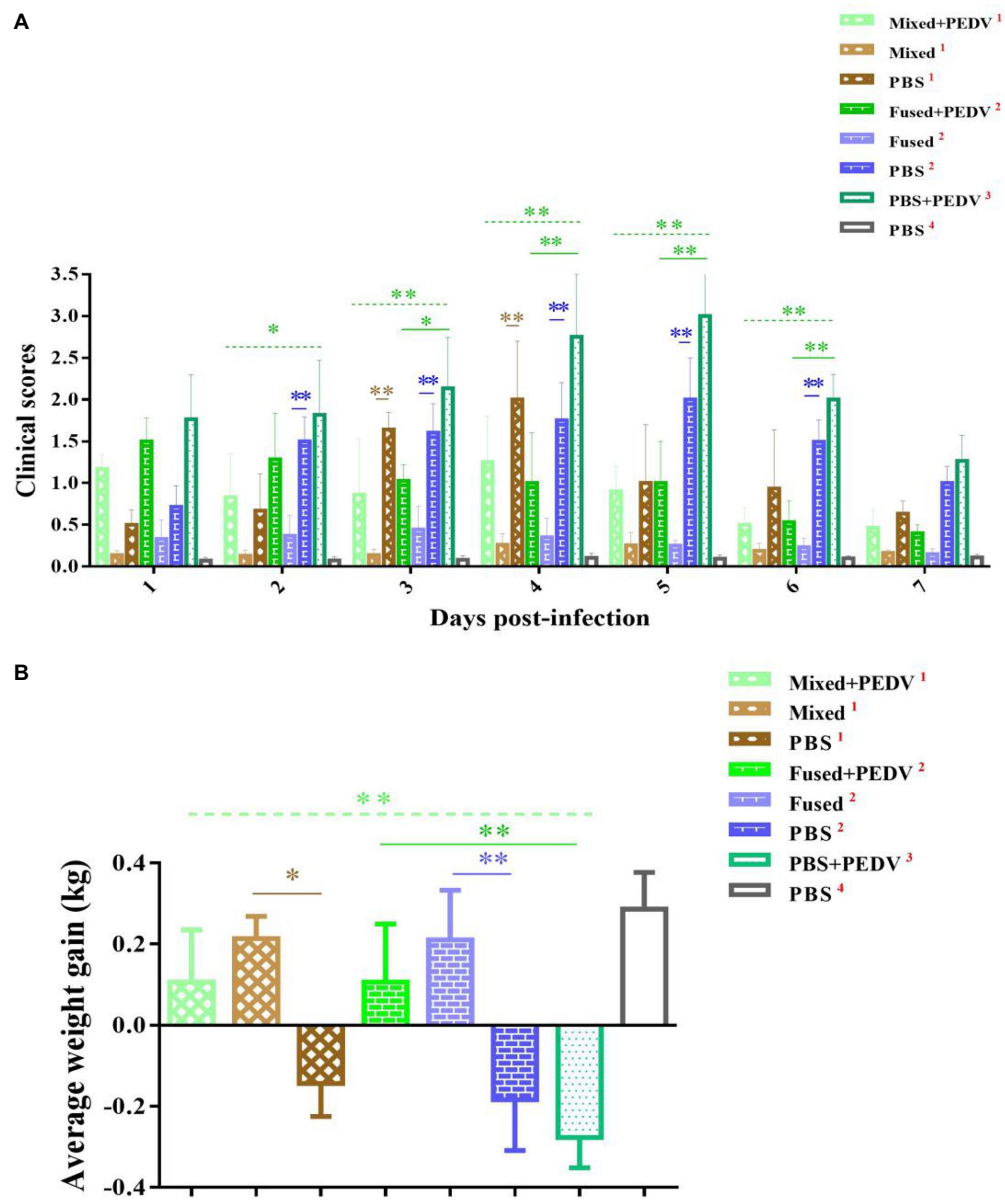
Clinical symptom scores and weight change of piglets. **(A)** Clinical symptom scores of piglets in different groups. **(B)** Average weight gain of piglets in different groups. *p < 0.05, **p < 0.01.

The challenge test using PEDV as an example was carried out after seven consecutive days of immunization; the piglets were grouped as shown in Figure 2. The clinical symptoms of the piglets in each group were observed after challenge and scored as shown in Table 1. Briefly, the piglets that were orally administered PBS followed by PEDV challenge (PBS + PEDV^3^) showed typical PEDV infection symptoms, including acute watery diarrhea, depression, and drowsiness, with the most severe symptoms observed between days 4 and 5 after challenge (Figure 6A). The immunized piglets (mixed+PEDV^1^ and fused+PEDV^2^) had slight diarrhea and a normal appetite and showed a significant difference from the PBS + PEDV^3^ group after 2 days (Figure 6A). None of the cohabiting piglets (mixed^1^ and fused^2^) developed diarrhea during the experiment and showed significant differences from unimmunized piglets (PBS^1^ and PBS^2^). In addition, before and after challenge, the weight of immunized piglets was significantly higher than that of unimmunized piglets (Figure 6B).

### Analysis on the Regularity of Virus Shedding Through Feces of Piglets

**Figure 7:**
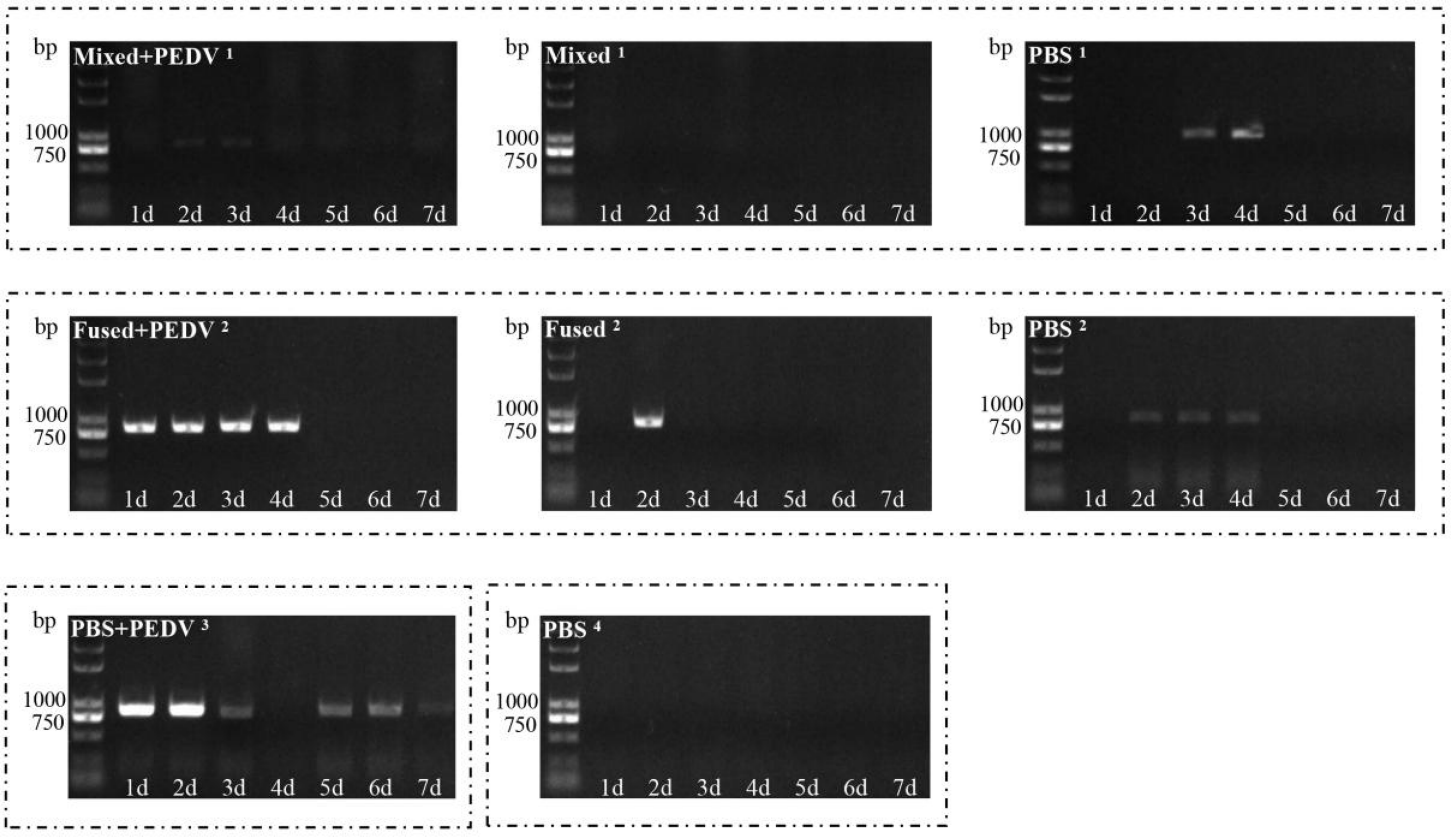
Detection of virus shedding through feces of piglets. PEDV RNA in anal swabs collected daily after grouping was detected using RT-PCR.

Anal swabs were collected from the piglets daily after grouping, and PEDV RNA in the anal swabs was detected using RT-PCR. The PBS + PEDV^3^ group began shedding the virus on day 1, which continued for the next few days (Figure 7). However, PEDV RNA was detected at 1–4 days in immunized piglets (mixed+PEDV^1^ and fused+PEDV^2^) and then disappeared (Figure 7). Among the cohabiting piglets, PEDV RNA was detected in unimmunized piglets (PBS^1^ and PBS^2^) 2–4 days after cohabitation; almost no fecal virus shedding was observed in the mixed^1^ and fused^2^ groups (Figure 7). Oral immunization with recombinant *Lactobacillus* inhibited PEDV shedding through the feces of piglets and reduced the infection risk of the cohabiting piglets. The inhibitory effect on fecal PEDV shedding in the mixed group was slightly stronger than that in the fused group.

### Macroscopic Examination and Histopathological Detection of Small Intestine in Piglets

**Figure 8:**
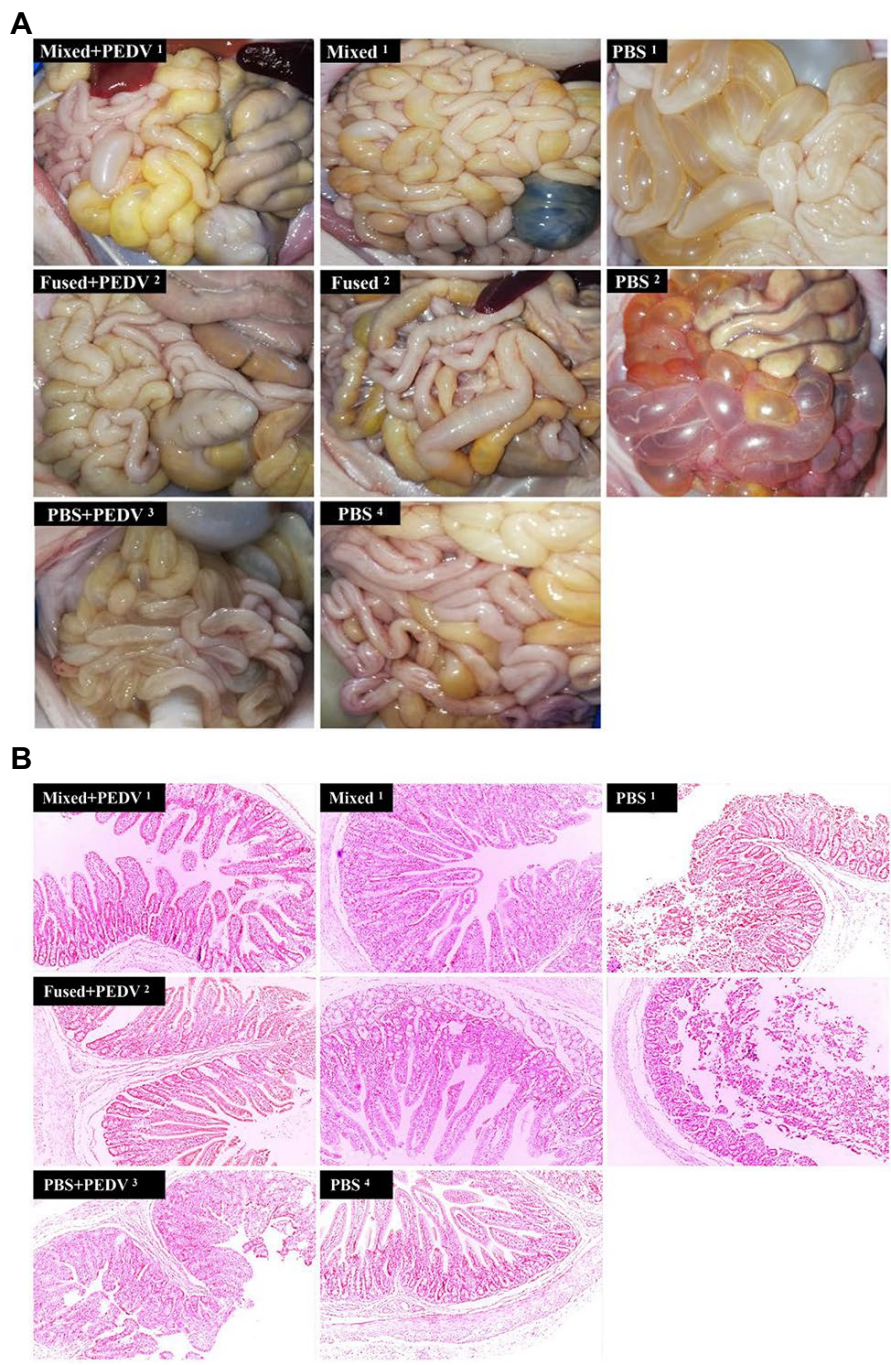
Intestinal macroscopic examination and histopathological observation of piglets. **(A)** Macroscopic examination of piglets in different groups. **(B)** Histopathological observation of jejunum of piglets in different groups. Original magnification: × 100.

All piglets were euthanized for macroscopic examination at 7 days after the challenge. Piglets in the PBS + PEDV^3^ group exhibited moderately thin and transparent intestinal walls in the small intestine, and large amounts of fluid accumulated in the intestinal lumen (Figure 8A). Cohabiting piglets (PBS^1^ and PBS^2^) exhibited similar or even more serious symptoms. In contrast, there were no observable changes in the small intestines of immunized piglets (mixed+PEDV^1^, fused+PEDV^2^, mixed^1^, and fused^2^) compared with those of the piglets in the PBS^4^ group (Figure 8A). Histopathological observations indicated that infection by PEDV caused extensive damage to the jejunum of piglets in the PBS + PEDV^3^ group, characterized by large amounts of fragmentation and shedding of intestinal villi (Figure 8B). Interestingly, the PBS^1^ and PBS^2^ groups exhibited more severe pathological damage to the jejunum. In contrast, damage to the jejunum of immunized piglets (mixed+PEDV^1^ and fused+PEDV^2^) was minor compared to that in the PBS + PEDV^3^ group. No pathological damage was observed in the piglets of the mixed^1^ and fused^2^ groups (Figure 8B).

## Discussion

By harnessing the mucosal immune system for vaccine development, sIgA and serum IgG can be induced to provide two layers of defense against mucosal pathogens (Thomas et al., 2015). Mucosal immunization can be achieved through oral administration, which is comparatively convenient to administer and is a far more effective route for directly stimulating intestinal sIgA responses (Neutra and Kozlowski, 2006; Sung et al., 2018). *Lactobacillus* is an ideal bacterial delivery system for oral vaccines (Rajoka et al., 2017; Chiabai et al., 2019; Liu et al., 2019; Natalia et al., 2019). Thus, we investigated the feasibility of using a multi-pathogen vaccine based on *Lactobacillus* spp. against porcine viral diarrhea.

We chose TGEV, PEDV, and PoRV, which are common pathogens of porcine viral diarrhea and often occur as mixed infections, to explore the feasibility of multi-pathogen vaccines. Antigens are key factors for the development of bacterial vector vaccines. The D site (378–395 amino acids) of the TGEV S protein has been identified as an important immune target for the host to neutralize the virus (Posthumus et al., 1990; Uer et al., 1991). To enhance immunogenicity, 6D fragments of the TGEV S protein were linked to each other to form a tandem gene, which was named as 6Ds. We previously showed that recombinant *Lactobacillus* expressing 6Ds induced mucosal and systemic immune responses in mice (Liu et al., 2011). Similarly, the COE and VP4 genes have been identified as the antigenic neutralizing epitopes of PEDV and PoRV (Duarte and Laude, 1994; Gilbert and Greenberg, 1998), respectively, and their effectiveness has been confirmed in the *Lactobacillus* expression system (Li et al., 2010; Hou et al., 2018). Therefore, we used the proteins 6Ds, COE, and VP4 as antigens.

To improve the expression of antigens and ensure their localization on the surface of *Lactobacillus*, the previously constructed expression vector pPG-T7g10-PPT (Song et al., 2014; containing the enhancer T7g10 fragment, HCE constitutive promoter, and PgsA anchor sequence), which expresses foreign antigens on the surface (Xu and Li, 2007; Liu et al., 2011; Yu et al., 2017; Zhao et al., 2017; Hou et al., 2018), was used in this study. We previously confirmed that recombinant *Lactobacillus* expressing a single antigen had a strong immune effect (Li et al., 2010; Liu et al., 2011; Hou et al., 2018). Research has suggested that combining multiple vaccines can have numerous positive effects against mixed multi-virus infections (Sánchez-Matamoros et al., 2019). Data on the immunity and safety afforded by such combined administration have been published for other vaccines in piglets (Jeong et al., 2016; Garcia-Morante et al., 2019; Sánchez-Matamoros et al., 2019). In this context, we constructed a recombinant *Lactobacillus* expressing antigen 6Ds, COE, and VP4, and immunity was evaluated following combined administration. To simplify the vaccine production schedules and reduce the cost of bacterial culture, another strategy involves the construction of a recombinant *Lactobacillus* expressing these three antigens in a fused manner. A previous study confirmed that direct fusion of antigens may lead to many undesirable outcomes, including misfolding of the fusion proteins, low yield in protein production, or impaired bioactivity (Amet et al., 2009). Therefore, the selection or rational design of linkers to join fusion proteins is important in recombinant fusion protein technology (Chen et al., 2012). Here, we chose a rigid linker to preserve the stability and bioactivity of the fusion proteins, and recombinant *Lactobacillus* pPG-T7g10-6Ds-COE-VP4/LC393 fused expressing the three antigens was constructed. Our results confirmed that the antigens 6Ds, COE, and VP4 were successfully expressed in recombinant *Lactobacillus* using pPG-T7g10-PPT as a vector. These antigens were expressed on the surface of the recombinant *Lactobacillus*.

Immune responses of newborn piglets induced by recombinant *Lactobacillus* were evaluated *via* oral immunization. Specific sIgA and IgG levels symbolize mucosal and humoral immune responses (Holmgren and Czerkinsky, 2005; Suzuki et al., 2017). In this study, anti-TGEV/PEDV/PoRV-specific sIgA and IgG were significantly increased in both the mixed and fused groups after immunization compared to those in the PBS group. The results showed that recombinant *L. casei* induced not only a specific mucosal immune response but also a specific humoral immune response against all three viruses in piglets, which is consistent with our previous results (Li et al., 2010; Liu et al., 2011; Hou et al., 2018). In addition, the anti-TGEV/PEDV/PoRV-specific sIgA and serum IgG in partial samples of the mixed group were slightly higher than those of the fused group, but the difference was not significant.

To determine whether the immune response of piglets induced by recombinant *Lactobacillus* could protect against viral infection, a challenge test was performed using PEDV as an example. Fecal-oral transmission is thought to be the main mode of PEDV transmission, and airborne transmission is considered as a potential route for dissemination (Alonso et al., 2014; Lin et al., 2016). The objectives of this study were to determine the efficacy of protecting piglets orally challenged with PEDV and to inhibit the horizontal spread of PEDV among piglets. For cohabiting piglets, the route of exposure to the virus is similar to that of natural infection rather than that from a high-dose oral challenge. Therefore, both oral challenge and cohabitation groups were examined in this study. Our results showed that recombinant *Lactobacillus* alleviated clinical symptoms, inhibited virus shedding, and reduced intestinal pathological damage in challenged piglets but could not prevent fecal virus shedding. The cohabiting piglets had no obvious symptoms, almost no fecal virus shedding, and no intestinal pathological damage, showing that recombinant *Lactobacillus* effectively protected cohabiting piglets. Thus, application of oral multi-pathogen recombinant *Lactobacillus* under field conditions may inhibit the horizontal transmission of PEDV and protect piglets from infection. Notably, protection of the mixed group was better than that of the fused group in terms of clinical symptoms and fecal virus shedding. The combination of multiple vaccines may have allowed the three recombinant *Lactobacillus* strains to play independent roles. In addition, because the specific sIgA and IgG levels induced by recombinant *Lactobacillus* against the three viruses were similar, the protective effect of these recombinant *Lactobacillus* against TGEV and PoRV may be similar to that of PEDV. To confirm this prediction, TGEV and PoRV challenge will be evaluated in further studies.

Overall, our results demonstrate that an oral multi-vaccine based on *Lactobacillus* can induce specific mucosal and humoral immune responses against multiple piglet diarrhea viruses and protect piglets from viral infection. *Lactobacillus*-based oral multi-vaccines show excellent potential as vaccines and should be further evaluated, which will have important implications for porcine management in highly convenient vaccination programs.

## Data Availability Statement

The original contributions presented in the study are included in the article/supplementary material, and further inquiries can be directed to the corresponding authors.

## Ethics Statement

The animal study was reviewed and approved by the Animal Experiment Ethics Committee of Northeast Agricultural University, China.

## Author Contributions

YL and LW conceived and designed the study. TG, CG, JH, XL, KX, XW, JL, HZ, and WC performed the experiments. ZS, YJ, XQ, and LT interpreted and analyzed the data. TG wrote the manuscript. All authors contributed to the article and approved the submitted version.

## Funding

This work was supported by the National Natural Science Foundation of China (31772779 and 31972718).

## Conflict of Interest

The authors declare that the research was conducted in the absence of any commercial or financial relationships that could be construed as a potential conflict of interest.

## Publisher’s Note

All claims expressed in this article are solely those of the authors and do not necessarily represent those of their affiliated organizations, or those of the publisher, the editors and the reviewers. Any product that may be evaluated in this article, or claim that may be made by its manufacturer, is not guaranteed or endorsed by the publisher.
